# Insulin-Egg Yolk Dispersions in Self Microemulsifying System

**DOI:** 10.4103/0250-474X.49092

**Published:** 2008

**Authors:** P. S. Singnurkar, S. K. Gidwani

**Affiliations:** USV Limited, B. S. D. Marg, Govandi, Mumbai-400 088, India

**Keywords:** Insulin, egg yolk, microemulsion, *in vitro* stability, oral delivery

## Abstract

Formulation of insulin into a microemulsion very often presents a physicochemical instability during their preparation and storage. In order to overcome this lack of stability and facilitate the handling of these colloidal systems, stabilization of insulin in presence of hydrophobic components of a microemulsion appears as the most promising strategy. The present paper reports the use of egg yolk for stabilization of insulin in self microemulsifying dispersions. Insulin loaded egg yolk self microemulsifying dispersions were prepared by lyophilization followed by dispersion into self microemulsifying vehicle. The physicochemical characterization of selfmicroemulsifying dispersions includes such as insulin encapsulation efficiency, *in vitro* stability of insulin in presence of proteolytic enzymes and *in vitro* release. The biological activity of insulin from the dispersion was estimated by enzyme-linked immunosorbant assay and *in vivo* using Wistar diabetic rats. The particle size ranged 1.023±0.316 μm in diameter and insulin encapsulation efficiency was 98.2±0.9 %. Insulin hydrophobic self microemulsifying dispersions suppressed insulin release in pH 7.4 phosphate buffer and shown to protect insulin from enzymatic degradation *in vitro* in presence of chymotripsin. Egg yolk encapsulated insulin was bioactive, demonstrated through both *in vivo* and *in vitro*.

Liquid emulsions have been used to deliver proteins and peptide orally. Emulsions are thought to protect the drug from chemical and enzymatic breakdown in the intestinal lumen. Drug absorption enhancement is dependent on the type of emulsifying agent, particle size of the dispersed phase, pH, solubility of drug and the type of lipid phase used. Water-in-oil microemulsions have been shown to enhance oral bioavailability of proteins and peptides^1^,^2^. The lipid phase of microemulsion composed of medium chain fatty acid triglycerides increased the bioavailability of muramyl dipeptide analog^3^. However, the major concern is the loss of biological activity caused by protein agglomeration^4^.

Egg yolk is one of the components of egg which mainly contains proteins, carbohydrates, lipids, minerals and water. It has excellent emulsifying properties and the phospholipids extracted from the egg yolk are mainly been utilized as emulsifier and in liposome preparations. Effect of egg yolk as penetration enhancer and to improve the stability of protein drugs is relatively less studied. Especially for insulin no reference was found from the literature search for use as penetration enhancer or to improve the stability of formulations for oral delivery^5^. The egg yolk contains 15.7-16.6% protein, 31.8-35.5% lipid, 0.2-1.0% carbohydrate and 1.1% ash^6^. Egg yolk is the major source for the production of purified phospholipids. The major fatty acids in egg yolk triglycerides are oleic acid (C18:1), palmitic acid (C16:0), linoleic acid (C18:2) and stearic acid (C18:0)^7^. Fatty acids like myristic acid (C14:0) and others are found only in trace amounts. Cholesterol contributes about 1.6% of raw egg yolk and about 5% of egg yolk lipids. Free cholesterol is about 84% of the total cholesterol with the remaining 16% being cholesterol ester^7^.

In this study, we tried to evaluate the combined use of egg yolk and self microemulsifying system to improve the oral bioavailability and stability of insulin. The process for the preparation of delivery system greatly influence the encapsulation efficiency and stability of the protein drug, especially when protein molecule comes in contact with hydrophobic surfaces, air-water interfaces, shear stress, temperature and organic solvents. In addition, size of the particles has been shown to have influence on the oral absorption^8^–^10^. Insulin-egg yolk dispersions were prepared by lyophilization followed by dispersing it into self microemulsifying solution.

## MATERIALS AND METHODS

Human insulin of Recombinant DNA origin was procured from Shreya Health Care, Mumbai. Tris hydroxymethylaminomethane (Tris buffer), m-cresol, hydrochloric acid 36% (HCl), sodium hydroxide pellets (NaOH) and anhydrous zinc oxide were all purchased from Merck Ltd., Mumbai. Egg yolk (Hen), propylene glycol, transcutol^®^ and glyceryl mono-linoleate were obtained from Gattefosse^®^. Cremophore RH 40 was from BASF, α-tocopherol acetate from Sigma Aldrich; polysorbate 80 from ICI India; glycerine from S. D. Fine Chem, Boisar and human insulin ELISA kits were from Mercodia^®^.

### Preparation of human insulin-egg yolk dispersion (InsEY):

Yolk from the chicken eggs was separated by decantation and used for the preparation of solid dispersion. Human insulin solution in tris buffer pH 7.4 (10 mmol, 200 IU/ml) equivalent to 1000 IU were added to about 4 g solids of egg yolk (equivalent to 8.5 g of egg yolk liquid), respectively to get final human insulin content of 0.250 IU/mg. The resultant dispersion was mixed on a magnetic stirrer (Remi^®^, India) for 10 to 15 min. The dispersion was transferred to tubular glass vials and frozen at −40° for 3 h and lyophilized for 38 h at condenser temperature of −40° and pressure of 112×10^−3^ mbar. After complete drying vacuum was broken with nitrogen gas to purge the vials with nitrogen and capped with butyl stopper and aluminium seals.

### Preparation of self microemulsifying solution (SMEDS):

Self microemulsifying solutions were prepared by glyceryl mono-linoleate, 25.4% w/v, α tocopherol acetate, 0.1% w/v, cremophore RH 40, 34.6%; transcutol, 10.8% w/v; and propylene glycol, 22.2% w/v. The mixture was stirred for 30 min at room temperature to get clear solution.

### Preparation of self microemulsifying solution containing insulin-egg yolk (InsEY-SMEDS):

Lyophilized human insulin-egg yolk powder was dispersed in the above optimised microemulsion solution to get 40 IU/ml human insulin content and mixed for 2 h at room temperature (NMT 25°) to get opaque dispersion. The solutions were stored at 10-15° till use in a glass vials.

### Characterization of self microemulsifying dispersions:

The size of InsEY-SMEDS particles was determined by dynamic laser light scattering (Brookhaven Instruments BI 90 Particle sizer, Holtsvilee, New York) at room temperature. For particle size analysis the InsEY-SMEDS dispersion was diluted with tris buffer pH 5.5 to obtain desired obscuration.

The morphology of the InsEY-SMEDS particles was viewed using a conventional scanning electron microscope (JSM 5400, Joel, Japan) at an accelerating voltage of I5 kV. One drop of the InsEY-SMEDS dispersion was placed on a graphite surface. After air drying, the sample was coated with gold using Ion Sputter.

### Determination of encapsulated insulin:

InsEY after lyophilization or InsEY-SMEDS was dispersed in 4 ml of 0.01N HCl (equal to 3 IU of human insulin) and mixed for 5 min on vortex mixer (Remi^®^ India) and sonicated for 10 to 15 sec at 20° temperature. Then volume the solution was adjusted to 5 ml and filtered through 0.22 μm syringe disc filter (Ultipor^®^ N66, Pall life Sciences). The filtered solution was quantitatively analysed for human insulin by RP-HPLC equipped with UV/Vis detector (Agilent 1200 series RRLC^®^). A reversed phase C_18_, 1.8 μm, 4.6×50 mm column. The gradient system consisted of mobile phase-A, mixture of buffer and acetonitrile (82:18), and mobile phase-B, mixture of buffer and acetonitrile (50:50). The flow rate was 1 ml/min with oven temperature of 40° and detection wavelength at 214 nm. The dispersions obtained after lyophilization were dissolved in a mixture of 0.01N HCl and ethanol (99%) with solvent ratio of 4:1, respectively. The solution was filtered though 0.22 μm syringe disc filter and injected. The entrapment efficiency (%) was determined by human insulin content obtained as percentage of initial amount used in the formulation.

### Release of insulin from self microemulsifying dispersions:

The release rate of human insulin from InsEY-SMEDS was determined by suspending the dispersions (equal to 100 IU of human insulin) in 50 ml, pH 7.4 phosphate buffer USP or 0.1N HCl. During the experiment (4 h) samples were shaken horizontally in a constant temperature shaker (Remi^®^, India) at 37±1° and 50 stokes per minute. At scheduled time intervals, 1 ml of sample was removed and replaced with 1 ml of fresh release medium. The sample was ultracentrifuged at 22,000 rpm at 20° for 15 min, then the supernatant was collected and quantitatively analysed for human insulin content by RP-HPLC.

### Effect of proteolytic enzymes on insulin stability:

*In vitro* degradation of insulin from InsEY-SMEDS dispersions was studies in presence of α-chymotrypsin (51 U/mg). In this study aqueous dispersion of InsEY-SMEDS in 0.05M phosphate buffer pH 7.8 was incubated for 30 min with α-chymotrypsin at 37±1°. The final concentration of the insulin and the enzyme were 15 U/ml and 26 U/ml. Insulin solution served as control. After incubation, the reaction was terminated by addition of 0.5 ml of 0.1% TFA. Subsequently, 0.5 ml of ethanol 99% was added to each tube and sonicated for 10 to 15 sec. Then the volume was adjusted to get the human insulin concentration of 0.5 to 0.6 IU/ ml with a mixture of 0.01N HCl and ethanol (4:1). The insulin solution was filtered though 0.22 μm syringe disc filter. The filtered solution was quantitatively analyzed for human insulin content by RP-HPLC.

### Estimation of biological activity of entrapped human insulin:

ELISA technique was used to evaluate whether human insulin entrapped in InsEY-SMEDS dispersions is biologically active. InsEY-SMEDS dispersions equivalents to 3 units of human insulin were dissolved in a mixture of phosphate buffer pH 7.4 and ethanol 99% with solvent ratio of 4:1. The solution was mixed for 5 min and then sonicated for 15 sec and volume adjusted to 5 ml and filtered through 0.22 μm syringe disc filter. Second dilution was made by diluting 50 μl of filtrate to 200 ml with phosphate buffer pH 7.4 to final concentration of about 150 mU/l. An aliquot of sample was withdrawn from the solution and insulin content was analyzed by ELISA as per standard protocol by measuring the optical density at 450 nm using plate reader (BioRad^®^, model 680 microplate reader) and results were calculated using Microplate Analyst 3.0.2 software.

### Pharmacodynamic study in diabetic rats:

Diabetes was induced in male Wistar rats (200-280 g) by intraperitoneal injection of streptozocin (40 mg/kg) in citrate buffer pH 4.5 via peritoneum. The solution of streptozocin was aseptically prepared and filtered through a 0.22 μm membrane filter (Millex-GV, Millipore). Rats were considered diabetic when fasted plasma glucose levels were near and above 300 mg/dl.

Diabetic rats, fasted overnight for 10 h, were injected subcutaneously with a single dose of human insulin injection (Huminsulin-R^®^ 40 IU/ml Injection, Elly Lili) at a dose level of 2 IU/kg. Six rats were used for this treatment group.

InsEY-SMEDS formulation as described above and the placebo of InsEY-SMEDS formulation were administered intragastrically in a single dose to diabetic rats that had been fasted overnight for 10 h. Six rats were used for each treatment group. The dose of insulin used for intragastric administration was 20 IU/kg. After administration of dosage form, 0.5 ml of purified water was intragastrically administered.

Prior to and at specified time intervals over 12 h period blood samples were collected from retroorbital vein in capillary tubes for serum human insulin estimation and fluoride oxalate impregnated capillary tubes for glucose estimation. The plasma samples were analyzed for glucose content by glucose oxygenase method (Reagent: Pinnacle Biotechnology Ltd, Mumbai) and measuring the optical density at 505 nm (Start 21 Autoanalyser). The serum samples were analyzed for the insulin content by using ELISA technique (Mercodia^®^ Insulin ELISA kit). Optical density at 450 nm was measured within 30 min on microplate reader (BioRad^®^, Model 680) and human insulin concentration was calculated using Microplate Analysis 3.0.2 software.

Human insulin pharmacokinetic parameters such as C_max_, T_max_, T_½_ and AUC_0-t_ were estimated from the serum human insulin concentration versus time profile. Semilogarithmic plot of the serum human insulin concentration versus time was constructed for estimation of pharmacokinetic parameters such as elimination rate constant, K_e_ and plasma half life, T_½_.

Blood glucose concentrations were determined in triplicate prior to dosing and the mean concentration was considered as 100% level. All following concentration-time data were expressed as a fraction of the base line, considering the fact that blood glucose concentrations over 12 h following intragastric administration of placebo nanopartic1es to diabetic rats (control groups) were not significantly different from the base line (assuming a flat base line). The mean±S.D. of each concentration-time point in each treatment group was calculated and compared.

The mean of percent blood glucose concentrations at each time point was subtracted from 100% and the area above the percent blood glucose-time curve (AAC_0-12h_) were estimated by the trapezoidal rule reported by Touitou and Rubinstein absorption^11^.

## RESULTS AND DISCUSSION

**Fig. 1:**
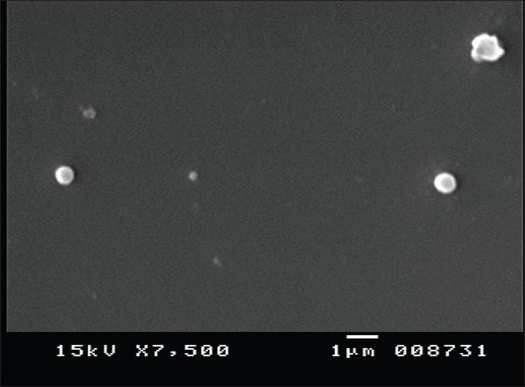
Photograph obtained by scanning electron microscopy of insulin-egg yolk particles. Scanning electron micrograph of human insulin-egg yolk particles at 10,000X, white spherical particles are observed.

Insulin-egg yolk dispersions after lyophilization showed characteristic of dense yellow cake formation with good dispersibility in purified water. The aqueous dispersions were opaque and pale yellow. The opaque appearance could be attributed to the emulsifying property of the egg yolk. Particle size of the SMEDS solution and InsEY-SMEDS showed very fine particle size with mean diameter of 0.387±0.052 μm for SMEDS solution. The self microemulsifying solution after addition into water resulted into formation of nanoemulsion. When InsEY was dispersed into SMEDS system and then added into water, increase in particle size to 1.023±0.316 μm was observed. It could be due to formation of dispersed particles of InsEY into SMEDS system and the presence of phospholipids and variety of fatty acids present in egg yolk. The SEM images (fig. 1) confirm that the dispersed particles are circular in shape and well dispersed and separated on the surface.

InsEY and InsEY-SMEDS dispersions were able to show high entrapment efficiency for human insulin and could be attributed to the process followed for the preparation of InsEY and InsEY-SMEDS dispersions.

**Fig. 2:**
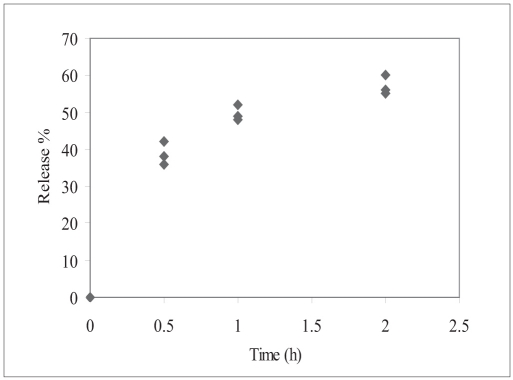
*In vitro* release behavior of insulin ♦ Percent cumulative human insulin release with time from insulin-egg yolk self microemulsifying dispersion in pH 7.4 phosphate buffer (n=3)

*In vitro* release of human insulin from InsEY-SMEDS dispersions in pH 7.4 phosphate buffer was evaluated. The dispersions were able to retard the release of insulin with initial bust effect. (fig. 2). The initial bust effect could be attributed to physical dispersion of insulin in egg yolk solids and releasing the insulin while forming spherical particles. There after little slower release of insulin was observed, could be due to the entrapment of insulin in the hydrophobic component, phospholipids of egg yolk and SMEDS microemulsion.

The *in vitro* stability of insulin in presence of proteolytic enzyme, α-chymotripsin, indicated that insulin was comparative more stable when formulated as InsEY-SMEDS dispersions as compared to insulin solution as a control. The results of residual human insulin content are depicted in Table 1. It can be seen that when insulin solutions were incubated with α-chymotripsin, ≤15% of human insulin remained undegraded after 30 min. InsEY-SMEDS incubated in the same medium, ≥51% of entrapped insulin remained undegraded over the same period. Each sample was analysed in triplicate.

**TABLE 1:** *IN VITRO* STABILITY OF INSULIN NANOPARTICLES IN PRESENCE OF α-CHYMOTRIPSIN

Formulation	% of undegraded human insulin
Human insulin crystals	17±2
InsEY-SMEDS formulation (InsEY-M)	51±2

Results are the mean of three observations±SD

Biological activity of entrapped human insulin in the InsEY-SMEDS formulations was estimated using ELISA technique. It can be seen that biological activity of human insulin from the InsEY-SMEDS dispersions was ≥97.2%. Samples were analysed in triplicate. No considerable loss of biological activity observed indicating the InsEY-SMEDS formulation components and the process of making the formulation preserved the integrity of human insulin.

**Fig. 3:**
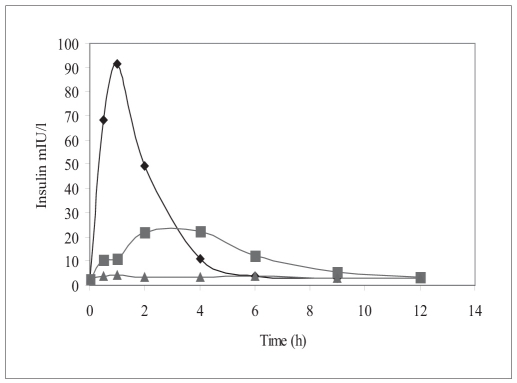
Serum human insulin versus time profile Serum human insulin levels after intragastric administration of 20 IU/kg InsEY-SMEDS dispersion (▪), Placebo formulation of InsEY-SMEDS equivalent to 20 IU/kg of insulin (▲), subcutaneous injection of 2 IU/kg human insulin solution (♦), n=6 per group.

*In vivo* studies in diabetic rats showed that human insulin is able to absorb from InsEY-SMEDS dispersions as indicated by serum human insulin concentrations over a period of 4 to 12 h as compared to placebo dispersions. However the serum human insulin concentrations levels were lower than the subcutaneous injection for insulin solution. Fig. 3 shows the serum human insulin concentration profile versus time for each group. The pharmacokinetic parameters such as C_max_, T_max_, AUC_0-t_, T_½_ were estimated for each group and the values observed are reported in Table 2. From area under human insulin concentration curves, AUC_0-12h_ for 20 IU/kg dose of InsEY-SMEDS formulation was lower than subcutaneous injection (SC. Inj.) of 2 IU/kg. The bioavailability of human insulin from subcutaneous injection was found to be diminished with subcutaneous injection of insulin solution while insulin from InsEY-SMEDS dispersions seemed to be continuing its absorption and hypoglycemic activity (fig. 3).

**TABLE 2:** PHARMACOKINETIC PARAMETERS FOR HUMAN INSULIN

Parameter	SC. Inj. Human insulin solution 2 IU/kg	Oral InsEY-SMEDS, 20 IU/kg
C_max_ (mIU/l)	91.5	22.3
T_max_ (h)	1	4
AUC_0-12h_ (mIU/l/h)	163.6	112.4
Ke	0.794	0.786
T_½_ (h)	0.872	0.881

Pharmacokinetic parameters are estimated from the each group of six male Wistar rats after subcutaneous injection of 2 IU/kg human insulin solution and oral administration of 20 IU/kg insulin self microemulsifying dispersion (InsEY-SMEDS).

**Fig. 4:**
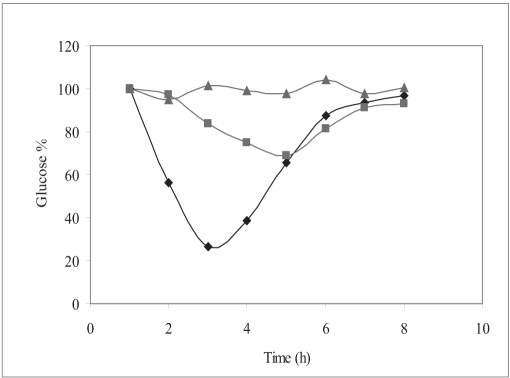
Mean plasma Glucose concentration (%) versus time curve Plasma glucose levels in percentage after intragastric administration of 20 IU/kg InsEY-SMEDS dispersion (▪), Placebo formulation of InsEY-SMEDS equivalent to 20 IU/kg of insulin (▲), subcutaneous injection of 2 IU/kg human insulin solution (♦), n=6 per group.

**Fig. 5:**
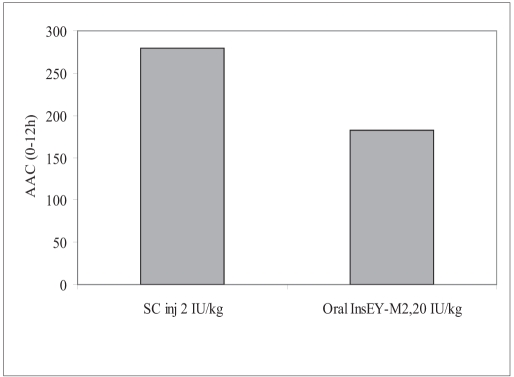
Plasma glucose AAC_0-12h_ Comparison of AAC_0-12h_ for intragastrically administered InsEY-SMEDS dispersion at 20 IU/kg with subcutaneous injection of human insulin solution at 2 IU/kg.

Hypoglycemic effect from InsEY-SMEDS dispersions was found to considerably higher than the placebo control group, however the extent of plasma glucose concentration reduction was highest with subcutaneous injection of human insulin solution. The reduction in the plasma glucose concentration was observed over the period of 4 to 12 h (fig. 4). Hypoglycemic effect of the 20 IU/kg dose was lower than 2 IU/kg subcutaneous injection of human insulin injection with respect to the extent of reduction in glucose concentration from base line (∼31% for 20 IU/kg, intragastrically administered human insulin InsEY-SMEDS and ∼73.5 % of baseline for 2 IU/kg subcutaneously injected human Insulin solution). Area above glucose concentration curves, AAC_0-12h_ calculated from these curves (fig. 4). AAC_0-12h_ of both the formulations depicted in fig. 5 show that the effect of 20 IU/kg dose of InsEY-SMEDS formulation was lower than subcutaneous injection of 2 IU/kg insulin, while the effect of the latter was diminished at 6 h after injection the former seemed to be continuing its hypoglycemic activity.

Insulin stability and biological activity was found to be enhanced in presence of egg yolk though insulin was formulated with hydrophobic components of SMEDS solutions. The lyophilization of insulin with egg yolk showed excellent entrapment of insulin into the formulation. The duration of *in vitro* insulin release was found to get extended beyond 3 h from InsEY-SMEDS dispersions, however estimation could not be performed for extended period due to degradation of insulin in the release medium at 37°. *In vivo* study in the streptozocin induced diabetic rats showed improvement in the oral absorption of insulin as indicated by serum human insulin AUC_0-12h_ and plasma blood glucose AAC_0-12h_.
